# Light-Triggered Carotenogenesis in *Myxococcus xanthus*: New Paradigms in Photosensory Signaling, Transduction and Gene Regulation

**DOI:** 10.3390/microorganisms9051067

**Published:** 2021-05-15

**Authors:** S. Padmanabhan, Antonio J. Monera-Girona, Ricardo Pérez-Castaño, Eva Bastida-Martínez, Elena Pajares-Martínez, Diego Bernal-Bernal, María Luisa Galbis-Martínez, María Carmen Polanco, Antonio A. Iniesta, Marta Fontes, Montserrat Elías-Arnanz

**Affiliations:** 1 Instituto de Química Física Rocasolano, Consejo Superior de Investigaciones Científicas, 28006 Madrid, Spain; diego.bernal@iqfr.csic.es; 2 Departamento de Genética y Microbiología, Área de Genética (Unidad Asociada al IQFR-CSIC), Facultad de Biología, Universidad de Murcia, 30100 Murcia, Spain; aj.moneragirona@um.es (A.J.M.-G.); ricardo.perez@um.es (R.P.-C.); eva.bastida@um.es (E.B.-M.); elena.pajares@um.es (E.P.-M.); mgalbis@um.es (M.L.G.-M.); mpolanco@um.es (M.C.P.); ainiesta@um.es (A.A.I.); mfontes@um.es (M.F.)

**Keywords:** photoreceptor, photosensitizer, photoregulation, singlet oxygen, plasmalogens, CarF, vitamin B_12_, CarH, ECF-sigma, CarD-CdnL

## Abstract

Myxobacteria are Gram-negative δ-proteobacteria found predominantly in terrestrial habitats and often brightly colored due to the biosynthesis of carotenoids. Carotenoids are lipophilic isoprenoid pigments that protect cells from damage and death by quenching highly reactive and toxic oxidative species, like singlet oxygen, generated upon growth under light. The model myxobacterium *Myxococcus xanthus* turns from yellow in the dark to red upon exposure to light because of the photoinduction of carotenoid biosynthesis. How light is sensed and transduced to bring about regulated carotenogenesis in order to combat photooxidative stress has been extensively investigated in *M. xanthus* using genetic, biochemical and high-resolution structural methods. These studies have unearthed new paradigms in bacterial light sensing, signal transduction and gene regulation, and have led to the discovery of prototypical members of widely distributed protein families with novel functions. Major advances have been made over the last decade in elucidating the molecular mechanisms underlying the light-dependent signaling and regulation of the transcriptional response leading to carotenogenesis in *M. xanthus*. This review aims to provide an up-to-date overview of these findings and their significance.

## 1. Introduction

Light is an important and ubiquitous signal in terrestrial and aquatic ecosystems, and the ability to sense, respond and adapt to light is crucial for most living organisms, including bacteria. Photosynthetic bacteria capture and convert light, an essential energy source, to chemical energy for cellular utilization, but light is also important for several other cellular processes in both phototrophic and non-phototrophic bacteria [1,2,3,4,5]. Thus, light is linked to many bacterial responses such as phototaxis, development, virulence, circadian rhythms and UV-induced DNA damage repair [4,5,6,7]. However, light can be harmful and cause cell damage and death. This stems from excitation of photosensitizing biomolecules, such as porphyrins, chlorophyll or flavins, to generate highly reactive oxygen species (ROS) like singlet oxygen (^1^O_2_), superoxides, peroxides and hydroxyl radicals that can destroy cellular DNA, protein and lipid components [4,8,9,10,11]. Consequently, bacteria have evolved ingenious mechanisms and machineries to mount a protective response to counter photooxidative stress.

A commonly used defense mechanism against photooxidative damage is through the biosynthesis of carotenoids, which quench and dissipate as heat the excess energy of ^1^O_2_ and other ROS produced upon illumination [4,8,9,11,12,13]. Carotenoids constitute a major class of lipophilic isoprenoid derivatives that are characterized by an extended, typically all-*trans*, conjugated polyene chain (usually C_40_ and some C_50_, C_45_ and C_30_ terpenes) with acyclic, monocyclic or bicyclic ends. Their oxygenated (hydroxy, aldehyde, keto, carboxyl, methoxy, epoxy, oxy and glycosidic) derivatives are called xanthophylls. Most carotenoids are richly colored (light yellow to deep red), since they absorb blue-violet light (400–500 nm range) owing to their extended conjugated double bonds that also determine the molecular conformation and reactivity [13]. Carotenoids also fulfill biological roles other than in photoprotection, such as in photosynthetic light harvesting, signaling and as precursors of photosensory molecules and hormones [13,14].

Carotenoid biosynthesis de novo occurs in all photosynthetic organisms (plants, algae or bacteria) and in many non-photosynthetic fungi, archaea and bacteria, whereas animals, save some strikingly few exceptions, do not synthesize carotenoids but obtain them exogenously [13,14]. Given that carotenoids are in the frontline of the defense against photooxidative stress, light and oxygen-related species like ^1^O_2_ are among the principal environmental factors involved in signaling and triggering carotenoid biosynthesis. This has been amply demonstrated in several studies from plants [15,16] and fungi [17,18] to bacteria [4,8,9,12]. Light-induced carotenogenesis and its regulation in the Gram-negative soil bacterium *M. xanthus* is undoubtedly one of the best studied and characterized among bacteria. We last reviewed this topic over a decade ago when many questions remained open [4,12]. Since then, considerable progress has been achieved largely from work in our group on the mechanistic, structural and photochemical aspects of light-regulated carotenogenesis in *M. xanthus*. Our work has uncovered new and large protein families, such as an entirely new class of photoreceptors with their novel mode of action that we specifically reviewed elsewhere [19,20,21,22]. It has also revealed the participation of “eukaryotic-like” proteins, including one found in *M. xanthus* and related myxobacteria, but absent in the vast majority of other bacteria, that turned out to be a long-sought human enzyme conserved across metazoa [23]. Our present review aims to provide a timely update of these findings, from signal reception and transduction to the transcriptional regulation underlying the photooxidative stress response and carotenoid biosynthesis in *M. xanthus*, and to discuss their mechanistic and evolutionary significance.

## 2. Biosynthesis of Carotenoids

**Figure 1:**
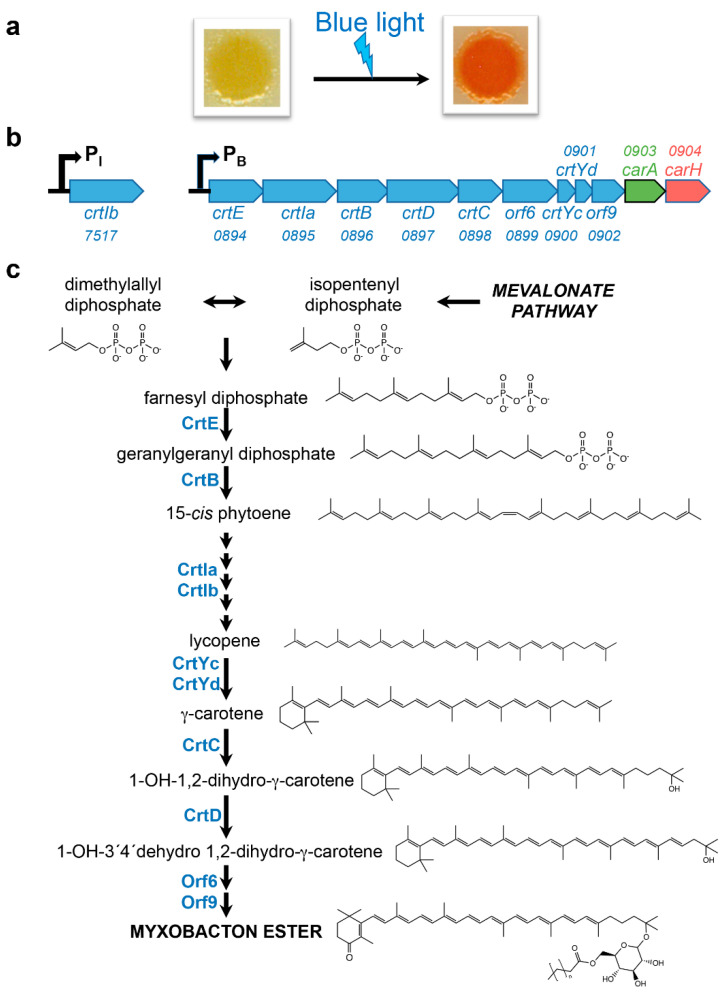
Carotenoid biosynthesis pathway and genes in *M. xanthus*. (**a**) *M. xanthus* colony color in the dark and in the light. Wild-type strains are yellow in the dark and red when exposed to blue light. (**b**) Structural genes for carotenogenesis characterized in *M. xanthus*. The *carB* locus encodes nine structural genes for carotenoid synthesis and two transcription regulatory factors, CarA and CarH, expressed from the primary σ^A^-dependent P_B_ promoter. The isolated structural *crtIb* gene is expressed from a promoter that depends on the ECF-σ factor CarQ (see text). The four-digit number above or below the corresponding gene indicates the original genome locus tag (MXAN_xxxx) of each of these genes. (**c**) Carotenoid synthesis pathway derived from the mevalonate (MVA) pathway in *M.* *xanthus*, with enzymes and products indicated.

Carotenoid biosynthesis occurs via a well-established and largely conserved pathway involving a number of genes and their products [13,14]. The pathway is considered to begin with the condensation of the universal five-carbon (C_5_) isoprenoid precursors isopentenyl diphosphate (IPP) and dimethylallyl diphosphate (DMAPP), themselves products of either the mevalonate (MVA) pathway (see Figure 1) or the non-mevalonate 2C-methyl-D-erythritol-4-phosphate (MEP) pathway [13,14,24]. Most bacteria and plastids are equipped with the MEP pathway, the MVA pathway is prevalent in animals, archaea, fungi and some bacteria including *M. xanthus* and the majority of myxobacteria, while plants and some select bacterial species use both pathways [13,14,24]. Condensation of IPP and DMAPP, the first committed and usually rate-controlling step in the core carotenoid biosynthesis pathway, produces geranylgeranyl diphosphate, two molecules of which then condense to generate the colorless C_40_ isoprenoid phytoene. A series of phytoene isomerization and desaturation steps generates the red carotenoid lycopene, from which carotenes and xanthophylls are produced in further desaturation, isomerization and hydroxylation reactions. Carotenoid biosynthesis and its regulation at levels from transcription, which is among the earliest and most crucial steps, to post-translation, degradation and feedback have been studied in many organisms [13,14]. Here, we discuss our current understanding of the *M. xanthus* carotenoid biosynthesis pathway, the structural and regulatory genes involved and how their transcription is induced and regulated.

## 3. A Brief History of Early Findings in *M. xanthus* Light-Induced Carotenogenesis

*M. xanthus* cells are yellow (Figure 1a) in the dark due to noncarotenoid, light-sensitive pigments that were identified and named DKxanthenes just fifteen years ago [25]. Four decades earlier, Burchard, Dworkin and coworkers reported that *M. xanthus* cells, when grown in the light, suffered photolysis or developed an orange/red color attributed to carotenoids and resisted photolysis, with the extent of illumination and growth phase determining the accumulation of carotenoids [26]. The action spectrum for photoinduction of carotenoids mirrored those for photolysis and for the absorption spectrum of protoporphyrin IX (PPIX), a hydrophobic cyclic tetrapyrrole and immediate precursor of heme in its biosynthesis, which accumulates in the *M. xanthus* cell membrane especially during stationary phase [26,27]. Photoinduction of carotenogenesis was maximal under blue light (405–410 nm), with lower maxima in the green light region (510–580 nm). Blue light excites the photosensitizer PPIX to ^3^PPIX, a very reactive high-energy triplet state that can directly cause cell damage or transfer its energy to other molecules [8,9]. Energy transfer from ^3^PPIX to molecular oxygen generates ^1^O_2_, an extremely reactive ROS that is relatively long-lived and diffusible in membrane environments [28]. Light-generated ^1^O_2_ was therefore proposed as the signal for carotenoid biosynthesis in *M. xanthus* [29], and later validated experimentally [30].

Two decades after these early findings, isolation and genetic analysis of *M. xanthus* spontaneous mutants, or ones generated by chemical, UV or Tn*5*-*lac* insertions, helped identify key genetic loci involved in light-induced carotenogenesis and established it as a transcriptional response [31,32,33,34]. The distinctive color change from yellow in the dark to red in the light (Figure 1a) due to light-induced carotenogenesis (the wild-type Car^+^ phenotype) provided a valuable visual tool for facile genetic analysis. This helped identify mutants that synthesize carotenoids constitutively (Car^C^) and are always orange/red, and these mutations mapped to two loci, *carA* and *carR*, which were inferred to encode negative regulators. On the other hand, mutants that never turn red in the light (Car^−^) were also identified and these mutations mapped to two loci encoding carotenogenic enzymes, or to various loci encoding putative positive regulators [32,33,34,35,36,37]. In these and subsequent studies, the loci were further mapped, epistatic relationships between them established, and the stage set for cloning, sequencing, gene expression assays and chemical analysis of carotenoids. This uncovered most of the structural and regulatory genes, and more recent biochemical, biophysical, genome-level and high-resolution structural analyses have provided profound insights into the molecular mechanisms underlying this light response.

## 4. Structural Genes Encoding *M. xanthus* Light-Induced Carotenoid Biosynthetic Enzymes

Genetic analysis, cloning and sequencing of the loci involved revealed that structural genes encoding the carotenoid synthesis enzymes (gene names usually prefixed *crt*) were located at the unlinked *carB* and *carC* loci [31,32,33,34,38,39,40]. The *carB* locus groups nine structural genes and two regulatory genes organized as *crtE-crtIa-crtB-crtD-crtC-orf6-crtYc-crtYd-orf9-carA-carH*, and the *carC* locus corresponds to a single gene, *crtIb* (Figure 1b). These annotations were based on analysis of sequence and of carotenoids accumulated in different mutants, as well as on heterologous expression in *E. coli* [33,38,39,40,41,42]. Genes *orf6* and *orf9* at the *carB* locus may also be structural ones whose functions remain to be established experimentally, while *carA* and *carH* encode transcription factors that regulate expression of *carB* genes (see below). The proposed carotenoid biosynthesis pathway for *M. xanthus* and the enzyme(s) involved in each step (Figure 1c) leads to synthesis of the final product, myxobacton ester, a monocyclic carotenoid with a keto group in the ring at one end of the molecule and a glycosyl group esterified to a straight-chain fatty acid at the other [43].

CrtE (geranylgeranyl pyrophosphate synthase), encoded by the first gene at the *carB* locus, catalyzes conversion of farnesyl diphosphate to geranylgeranyl diphosphate, two molecules of which condense to phytoene through the action of CrtB (phytoene synthase). The colorless phytoene is isomerized and transformed in four successive dehydrogenation steps to the red acyclic lycopene by the synergistic action of the CrtIa and CrtIb phytoene dehydrogenases [38,39,41]. Lycopene is cyclized at one end to monocyclic γ-carotene by the concerted action of CrtYc and CrtYd, members of the heterodimeric lycopene monocyclase family encoded by adjacent genes at *carB* [42]. The γ-carotene is subsequently hydroxylated by hydroxyneurosporene synthase (CrtC), desaturated by hydroxyneurosporene dehydrogenase (CrtD), and appended with a sugar moiety possibly by the action of the *orf6* gene product, a predicted glycosyltransferase. Finally, a putative acyltransferase encoded by *orf9* may act in myxobacton esterification [12]. Regulation of the carotenoid biosynthesis pathway can occur at the levels of transcription, post-transcription, modulation of enzyme activity through crosstalk and cooperation between them (such as the synergistic action of CrtIa and CrtIb mentioned above) and feedback regulation by the final carotenoid product and/or precursors. Moreover, how pathways intertwined with that for carotenogenesis, such as the MVA or the PPIX/heme biosynthesis pathways, are regulated can be important. Clearly, regulation at the transcription level is among the earliest and most crucial, and light-dependent regulation of transcription of *crtIb* and of the genes at *carB* has been intensely studied in *M. xanthus*.

## 5. Two Modes of Light Sensing and Signaling in *M. xanthus* Carotenogenesis

Since light triggers expression of *carB* and *crtIb*, understanding how light is sensed and converted to a cellular signal to mount the transcriptional response in *M. xanthus* is critical. In most living organisms including bacteria, the crucial task of sensing and transducing the light signal depends on photoreceptors, which are specialized proteins equipped with covalently or noncovalently bound light-sensing cofactors called chromophores. Photoreceptors have been classified into ten families thus far based on the specific chromophore and the protein photosensory domain [5,21,44,45,46,47]. One or more of these proteins occur in various bacteria, some are more widely distributed than others, and some occur even in species with minimal genomes or lifestyles that might suggest an absence of photoreceptors. Yet, surprisingly, given its well-established light response and a genome that is among the largest and most complex across bacteria, *M. xanthus* appeared to lack known photoreceptors. Consequently, blue light sensing through PPIX and the corresponding, rather convoluted, signaling pathway (discussed in Section 7) were considered as the sole mechanism to trigger carotenogenesis in *M. xanthus*. This changed about ten years ago with the discovery of the first member of an entirely new family of photoreceptors, which established a second light sensing and signaling mechanism that is simpler and more direct [20,21].

## 6. Direct Light Sensing, Signal Transduction and Gene Regulation by the B_12_-Based CarH Photoreceptor

Discovery of the more direct light-sensing/signaling pathway and of a new photoreceptor family emerged from studies of the two adjacent and most downstream genes of the *carB* cluster, *carA* and *carH,* whose expression is significantly enhanced in the light [20,21,38,40]. Whereas mutations at *carA* yielded a Car^C^ phenotype, linking it to a negative regulator [32,34], a *carH* deletion had no apparent effect [48], even though the corresponding gene products of comparable sizes (CarA: 288 residues; CarH: 299 residues) share ~48% similarity (~35% sequence identity) and a similar two-domain architecture [38,48]. In both proteins, the ~70-residue N-terminal region resembles the DNA-binding domain (DBD) of MerR family proteins [38], which are widespread transcription factors in bacteria that repress or activate gene expression in response to diverse environmental stimuli such as oxidative stress, heavy metals or antibiotics [49]. MerR proteins bind as dimers via their winged-helix DNA binding domains to specific (pseudo)palindromic sites located within or overlapping their target primary σ^A^-dependent promoters, and binding of a ligand (metal/drug) to a C-terminal module or oxidation of a redox center in it, enables these proteins to modulate transcription [49,50,51,52]. Notably, the ~200-residue C-terminal domain in CarA and CarH resembles a domain that binds to methylcobalamin (MeCbl) [48], one of the two biological forms of vitamin B_1__2_, in the methionine biosynthesis enzyme MetH, a methionine synthase. The MetH B_12_-binding domain (B_12_-BD) houses a signature motif, E/DxHx_2_Gx_41_SxTx_22-27_GG, whose His supplies the lower axial ligand in the so-called base-off/His-on binding to B_1__2_ [53]. Prior to CarA and CarH, such B_12_-BDs were reported only in enzymes using B_1__2_ as a cofactor [54,55]. The combination of a B_12_-BD and a DBD in CarA and CarH was therefore unprecedented and hinted at a pair of unusual transcription factor paralogs. Identifying a role for B_12_ and its mode of action, however, turned out to be less than straightforward.

### 6.1. CarH and Vitamin B_12_ Regulate Light-Induced Expression of Carotenoid Genes

Mapping the transcription start site at the *carB* locus identified a light-inducible primary σ^A^-dependent promoter, P_B_, with a consensus TTGACA –35 element and a less conserved TACCTC –10 element [38], which was recognized by σ^A^-bound RNA polymerase (RNAP) in vitro [56]. CarA was found to dimerize via its B_12_-BD [20,57] and use its N-terminal DBD (which indeed structurally resembles MerR DBDs [58]) to bind cooperatively, as two dimers, to a large ~55-bp DNA segment at the P_B_ promoter region (from positions –70 to –19 relative to the transcription start site) [56,57,59]. Since the operator overlaps with the –35 P_B_ promoter element, CarA binding can block promoter access to RNAP-σ^A^ and repress transcription [56]. Surprisingly, even though CarA could bind B_12_, consistent with the presence of a canonical B_12_-binding motif at its C-terminal domain, it neither required B_12_ for operator binding in vitro nor did mutating key residues in its B_12_-binding motif impair P_B_ repression in the dark in vivo [60]. Key to unmasking the role of CarH was the finding that the Car^C^ phenotype caused by deleting *carA* could be reverted to wild-type behavior upon addition of exogenous vitamin B_12_ to the growth medium (*M. xanthus* takes up and assimilates B_12_ but cannot synthesize it de novo) [60]. CarH was shown to orchestrate this B_12_-dependent repression of P_B_ in vivo and its relief in the light, and this activity of CarH required an intact CarA operator [60]. Thus, CarA and CarH both target the same operator at P_B_ to control light-induced expression of all but one of the carotenogenic genes in *M. xanthus*, but only CarH absolutely required B_12_ for activity. These findings not only established a functional link between B_12_ and CarH but also revealed a novel facet of this vitamin: its use in a cellular light response.

### 6.2. Molecular Architecture and Mode of Action of the B_12_-Based CarH Photoreceptor

Answers to what specific B_12_ form was required by CarH and its molecular mechanism of action, as well as why and how CarH differs from its paralog CarA, began to emerge with a seminal study ten years ago [20]. CarA and CarH remained the first and only known transcription factors with a B_12_-binding motif until homologs of unknown function were revealed in bacterial genomes covering a vast taxonomical range beyond myxobacteria [20,21]. This allowed comparative studies and better molecular understanding of these proteins. Whereas CarH has thus far resisted purification in the native form, two of its homologs from bacteria unrelated to *M. xanthus* have been purifiable in a native soluble form and could therefore be well-characterized in vitro. Both homologs turned out to be B_12_-dependent like CarH. Studies of the homolog in the Gram-negative *Thermus thermophilus*, CarH_Tt_, yielded valuable biochemical [20], structural [19] and photochemical insights [61,62,63] that were further extended with CarH_Bm_, the homolog in the Gram-positive *Bacillus megaterium* [64,65]. These findings, reviewed elsewhere [21,22], are briefly highlighted here.

**Figure 2:**
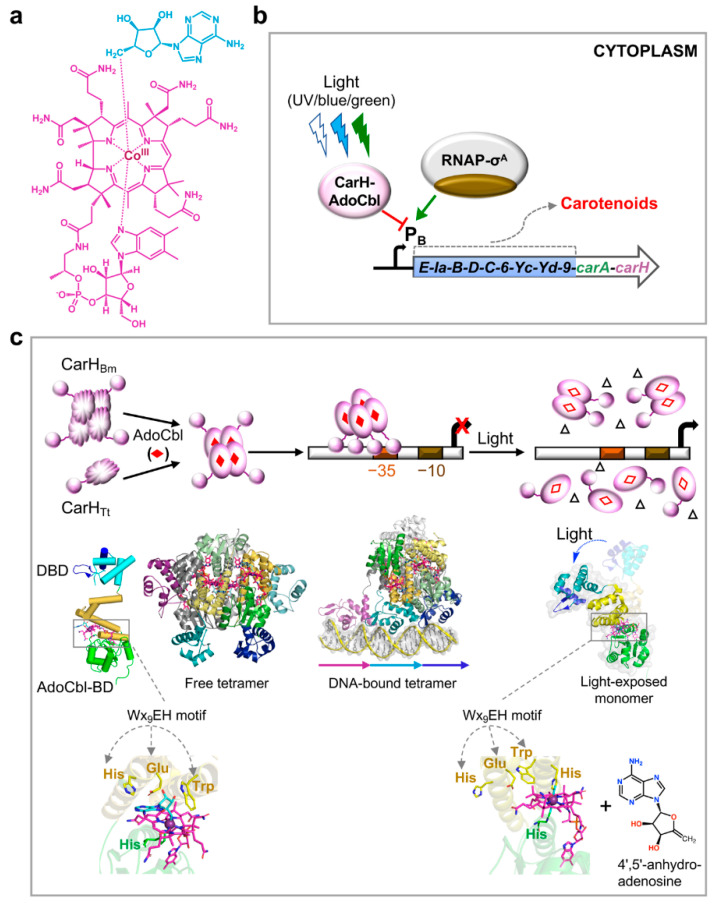
Light sensing and gene regulation by the B_12_-based CarH photoreceptor. (**a**) Chemical structure of AdoCbl, the light-sensing chromophore of the CarH photoreceptor, with the upper axial 5′-deoxyadenosyl group in cyan and the rest of the molecule in magenta as depicted in the structures below. (**b**) CarH-mediated regulation at P_B_. In the dark, AdoCbl-bound CarH binds to its operator at P_B_ to block access to RNAP-σ^A^ and repress transcription; and light (UV, blue or green) inactivates CarH to prevent its binding to operator, allowing P_B_ access to RNAP-σ^A^ and transcription initiation. (**c**) Molecular mechanism of CarH-mediated regulation at P_B_. AdoCbl (filled red diamonds) bind to apo form monomers (CarH_Tt_) or molten globule tetramers (CarH_Bm_) to produce active, properly folded, compact tetramers that bind in the dark to an operator overlapping with a σ^A^-dependent promoter (shown for –35 region but can be –10 or both) and thereby block transcription. UV, blue or green light photolyzes CarH-bound AdoCbl and disrupts DNA-bound tetramers to monomers (CarH_Tt_) or dimers (CarH_Bm_) that retain photolyzed AdoCbl (open red diamonds), leading to loss of operator binding and transcription. Upon photolysis, the upper ligand of AdoCbl is released as 4′-5′-anhydroadenosine (open triangles). Structures for AdoCbl-bound CarH_Tt_ tetramer, free and DNA-bound, and for the light-exposed monomer are shown below. The protomer structure (left) with the DBD in cyan (recognition helix and wing, dark blue) and the AdoCbl-BD, with its four-helix bundle subdomain in golden, Rossmann fold subdomain in green, AdoCbl colored as in (**a**). PDB accession codes; 5C8D (tetramer in the dark), 5C8E (DNA-bound tetramer in the dark), 5C8F (light-exposed monomer). Below are close-ups of the Trp, Glu and His of the Wx_9_EH motif capping the Ado group of AdoCbl in CarH_Tt_, with the lower axial His (green) in the dark state (left) and light-exposed (right) state. In the latter, a His adjacent to the Trp in the Wx_9_EH motif becomes the upper axial ligand in a bis-His linkage. The 4′-5′-anhydroadenosine product of AdoCbl-CarH_Tt_ photolysis is also shown (far right bottom).

The specific B_12_ form required in CarH-mediated regulation of light-induced carotenogenesis in *M. xanthus* was established as 5’-deoxyadenosylcobalamin (AdoCbl) or coenzyme B_12_ (Figure 2a), which binds to the CarH C-terminal domain and directs its oligomerization and function [20]. AdoCbl is a complex organometallic molecule with a central cobalt, generally Co^3+^/Co(III), coordinated to: (a) four equatorial pyrrolic nitrogens of the corrin ring; (b) a lower axial nitrogen from the 5,6-dimethylbenzimidazole (DMB) moiety linked to the corrin ring (so-called base-on or DMB-on conformation), or histidine from the B_12_-binding motif in a protein (base-off/His-on binding, mentioned earlier); (c) an upper axial 5′-deoxyadenosyl (Ado) group; this upper ligand is methyl (Me) in MeCbl or cyano (CN) in vitamin B_12_, a nonbiological form. The Co-C bond to an alkyl carbon in AdoCbl or MeCbl confers some unique and useful chemical properties. Its enzyme-catalyzed cleavage, which enables the use of AdoCbl in mutases, dehydratases, deaminases and ribonucleotide reductases and of MeCbl in methyltransferases, has been extensively studied and reviewed elsewhere [54,55]. Cleavage of the Co-C bond, by near-UV and visible light of wavelengths <530 nm, also underlies the use of AdoCbl as a chromophore for light sensing and response by CarH proteins (Figure 2b,c), which now represent a separate, large and widespread photoreceptor family among the ten currently known [19,20,21,22,61,62,63].

Studies of CarH_Tt_ and CarH_Bm_ revealed that light-dependent regulation of transcription relies on modulation of their oligomeric state by AdoCbl and light (Figure 2c) [19,20,21,22,64,65,66]. AdoCbl-free apoCarH_Tt_ is a monomer and apoCarH_Bm_ is a loosely folded molten globule tetramer, and both bind poorly to operator DNA. Both proteins form AdoCbl-bound tetramers in the dark that bind tightly to a large operator, which overlaps with the target gene promoter, to thwart access to RNAP-σ^A^ and block transcription; in the light, cleavage of the Co-C bond frees the upper axial Ado group and provokes tetramer disassembly to photolyzed CarH_Tt_ monomers or CarH_Bm_ dimers that detach from the operator to allow RNAP-σ^A^ binding and transcription initiation [19,20,64]. Cleavage of the AdoCbl chromophore with release of the Ado group is irreversible, in contrast to the usually reversible light-induced molecular changes observed with other photoreceptor chromophores [44,45,47], suggesting that there may be pathways to recover and reuse the chromophore that remain to be identified. Available data suggest that, like CarH_Tt_, CarH is a monomer in the light-exposed AdoCbl-bound and apo forms, and that the dark AdoCbl-bound form is oligomeric but its stoichiometry remains to be defined [20].

Crystal structures of the AdoCbl-CarH_Tt_ tetramer, free or DNA-bound, and of the light-exposed AdoCbl-CarH_Tt_ monomer provided detailed molecular snapshots of CarH architecture and its light-dependent mechanism of action [19]. It confirmed the two-domain CarH modular architecture, with a MerR/CarA-like winged-helix N-terminal DBD connected by a flexible, disordered linker to a C-terminal AdoCbl-binding domain (hereafter AdoCbl-BD), in which AdoCbl is sandwiched between a four-helix bundle and a Rossmann fold subdomain (Figure 2c). The AdoCbl-BD is structurally similar to the MetH MeCbl-binding domain but has a critical Wx_9_EH motif in the four-helix bundle that caps the upper axial Ado, which is absent in MetH (Figure 2c). In addition to the classic ExHx_2_Gx_41_SxV/Tx_22-27_GG B_12_-binding motif, the Wx_9_EH motif is absolutely conserved in all CarH homologs studied thus far and its critical role in AdoCbl-binding and function has been experimentally demonstrated [19,64]. Thus, absence of the motif in CarA can largely account for its B_12_-independent activity. Indeed, the presence of both signature motifs defines CarH homologs, and several hundreds of these now assigned from genome data are broadly distributed across diverse bacterial taxa [21,22]. The dark state AdoCbl-CarH_Tt_ tetramer is a dimer of two dimers, each of which is itself assembled by head-to-tail packing of two monomers via their AdoCbl-BD, with Trp of the Wx_9_EH motif playing a crucial role. Since tetramer formation is very favorable, dimers are detected only by disruption of the dimer-dimer interface, such as by mutation [19,20,66]. In this unusual AdoCbl-CarH_Tt_ tetramer assembly, the DBDs of neighbouring monomers point away from each other on the tetramer surface, which results in an unexpected DNA binding mode, wherein one DBD contacts a 11-bp direct repeat (DR) with a consensus nAnn**T**nn**ACA**n sequence (*n* = any base). Hence, it differs from the typical (pseudo)palindromic DNA sites of MerR proteins, yet it conserves most of the DNA contacts. Whereas three such tandem 11-bp DRs comprise the CarH_Tt_ operator, four of these constitute the CarH_Bm_ operator [19,64] and, likely, the ~55-bp *M. xanthus* CarH operator [20], suggesting a notable DNA-binding plasticity. Comparing the tetramer structure with that determined for the photolyzed CarH_Tt_ monomer yielded molecular insights into light-induced tetramer collapse and loss of DNA binding. The light-exposed form revealed bound photolyzed AdoCbl (without the upper axial Ado) and a large shift (>8 Å) of the four-helix bundle relative to the Rossmann fold (Figure 2c), which disrupts the head-to-tail dimer interface, and thereby the tetramer, leading to loss of DNA binding.

The photochemistry of CarH_Tt_-bound AdoCbl examined by analyzing photolysis products [61], by ultrafast spectroscopy [62,63] and by theoretical calculations [67] suggested it may differ significantly from that established for free or enzyme-bound AdoCbl. Photolytic cleavage of free AdoCbl, often a model for that in AdoCbl-dependent enzymes, is homolytic and generates reactive cob(II)alamin and Ado• radical species that rapidly react to yield specific products depending on the presence or otherwise of molecular oxygen [21,61,68]. In AdoCbl-dependent enzymes, which also rely on homolytic Co-C bond cleavage, the cob(II)alamin and Ado• radical species are generated in carefully controlled protein environments to ensure the difficult radical-based enzyme action and cofactor recovery, and to simultaneously limit enzyme damage and unwanted side reactions [69]. Since CarH controls a cell response (carotenogenesis) precisely to combat reactive ROS like ^1^O_2_, its use of an AdoCbl chromophore with an underlying irreversible photolytic Co-C cleavage that releases reactive radicals seemed paradoxical. Remarkably, CarH appears to resolve this problem by altering AdoCbl photochemistry for its safe use as a photoreceptor chromophore. It was found that photolysis of CarH_Tt_-bound AdoCbl avoids release of Ado• radicals by generating 4′,5′-anhydroadenosine, a harmless product undetected upon cleavage of free or enzyme-bound AdoCbl ([61]; Figure 2c). Based on ultrafast spectroscopy data, it has been proposed that CarH enables an unprecedented heterolytic cleavage of the AdoCbl Co–C bond to bypass radical formation and release [62] or stabilizes an excited state long enough to ensure the reactions that yield the 4′,5′-anhydroadenosine product [63]. The molecular mechanism for how CarH alters AdoCbl photochemistry is still unclear. It has been speculated that molecular oxygen and residues around the Ado group, notably of the Wx_9_EH motif, may be important.

## 7. Blue Light Sensing, Signaling and Gene Regulation in the B_12_-Independent Pathway

### 7.1. Light Is Perceived through Photoexcitation of PPIX, Which Leads to ^1^O_2_ Production

Although the blue light-PPIX sensing and signaling mechanism to induce carotenogenesis in *M. xanthus* was the first to be identified, it is also the more complex one. Genetic evidence for the role of PPIX came from analysis of *M. xanthus* strains bearing specific deletions of genes in the heme biosynthetic pathway that resulted in elimination or overproduction of endogenous PPIX [30]. Thus, a strain with a deletion of *hemB*, which encodes an early enzyme in the heme biosynthetic pathway was Car^−^, and the Car^+^ phenotype could be restored by supplying PPIX exogenously. On the other hand, a strain with a deletion of *hemH*, whose product incorporates ferrous iron into PPIX in the final step of the heme biosynthetic pathway, exhibited a markedly enhanced light-induced carotenogenesis. The light response thus requires PPIX and correlates with the photosensitizer levels. The need for blue light and PPIX to induce carotenogenesis could be bypassed using the phenothiazinium dye methylene blue and red light, which also generates ^1^O_2_, and was suppressed by ^1^O_2_ quenchers [30]. The blue light signal is thus transduced via PPIX to ^1^O_2_ and then relayed via a recently identified (and unprecedented) mechanism, whose molecular details continue to be unfurled.

### 7.2. CarF and Plasmalogen Lipids in M. xanthus Blue Light-PPIX-^1^O_2_ Signaling

**Figure 3:**
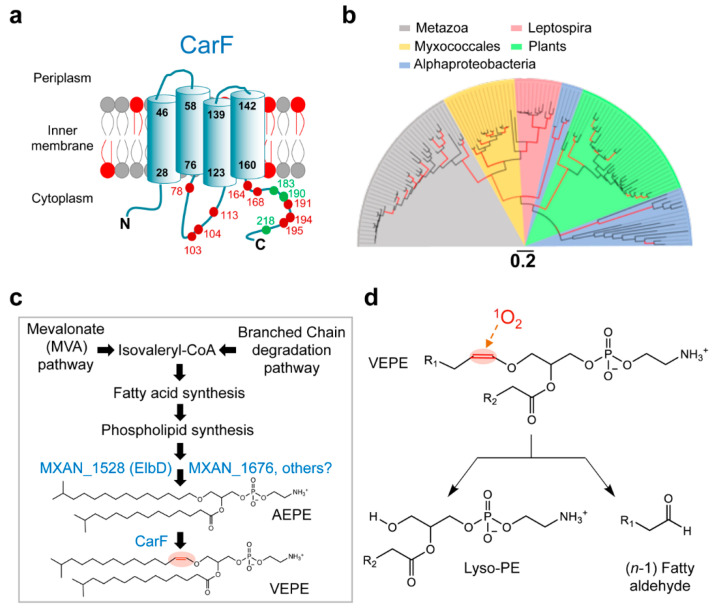
*M. xanthus* CarF and plasmalogen synthesis. (**a**) Cartoon representation of the *M. xanthus* CarF protein depicting its experimentally established membrane topology with four transmembrane helices (delimiting residues of each helix numbered in black). Numbered dots correspond to the 12 histidines in CarF. Nine of these (in red) are essential for CarF function but not the rest (in green); all nine essential histidines are conserved in animal CarF homologs, and all of these except His113 in plant homologs. The inner membrane plasmalogens are depicted in red. (**b**) Maximum-likelihood unrooted phylogenetic tree based on selected CarF homologs in metazoa, bacteria and plants (distributed in different colored sectors as indicated; branches in red, ≥75% confidence values from 200 bootstrap replicates; scale bar, number of substitutions per residue). (**c**) The *M. xanthus* plasmalogen biosynthesis pathway highlighting the early pathways and the final step, in which CarF mediates the desaturation that converts its alkyl ether lipid AEPE (1-*O*-(13-methyltetradecyl)-2-(13-methyltetradecanoyl)-glycero-3-phosphatidylethanolamine), to the plasmalogen VEPE (1-*O*-(13-methyl-1-*Z*-tetradecenyl)-2- (13-methyltetradecanoyl)-glycero-3-phosphatidylethanolamine). (**d**) Blue light-PPIX generated ^1^O_2_ cleaves the vinyl ether bond of plasmalogens (VEPE) to yield a lyso-PE (2-monoacylglycerophosphoethanolamine) and an (*n-1*) fatty aldehyde (and formic acid, not shown).

Signaling by ^1^O_2_ produced by blue-light photoexcitation of PPIX absolutely requires CarF [30], which was found in an analysis of Tn*5*-*lac* mutants and mapped to a locus unlinked to those previously identified in *M. xanthus* [70]. CarF is a 281-residue membrane protein with a four transmembrane-helix topology (Figure 3a), and its expression is not light-dependent [70,71]. Sequence homology searches [23] revealed that bacterial CarF-like proteins are present only in myxobacteria and a few Leptospiraceae and Alphaproteobacteria but, intriguingly, they are widespread in animals (invertebrates and vertebrates including humans, where the homolog is named TMEM189 or Kua [72]) and in plants. Protein phylogenetic analysis clearly indicated that CarF homologs from animals and from Leptospira are more related to those in *M. xanthus* and other myxobacteria, and those from Alphaproteobacteria and plants group together and are less related to CarF (Figure 3b). Until very recently their functions were largely unknown, except for the fact that CarF was required in the *M. xanthus* light response, and that a plant CarF homolog was a chloroplast fatty acid desaturase (FAD4) that generates an unusual trans double bond in the *sn*-2 acyl carbon chain [73].

A notable feature of CarF is its many (12) histidines, all cytoplasmic, with nine being essential for function ([23]; Figure 3a). The distribution of these histidines, some as HxxxH and HxxHH motifs, resembles that in membrane-associated diiron fatty acid desaturases and hydroxylases of otherwise low overall sequence similarity to CarF [23,70,71,72]. Hence, these observations hinted that CarF might be a fatty acid desaturase, like FAD4, but probably of a different kind, given that FAD4 lacks one of the crucial histidines in CarF [23].

The exact function of CarF and its role in *M. xanthus* light-induced carotenogenesis has only now been established [23]. It was discovered that CarF and its homologs in animals from worm and fly to fish, mouse and human, but not those in plants, correspond to the long-sought plasmanylethanolamine desaturase (now named PEDS1). This enzyme converts plasmanylethanolamine or alkyl ether phosphatidylethanolamine (glycerophospholipids with the *sn*-1 hydrocarbon chain linked by an ether bond instead of the typical ester bond; hereafter, AEPE) to plasmenylethanolamine, the alkenyl or vinyl ether phosphatidylethanolamine (hereafter, VEPE; Figure 3c). VEPE and analogs with choline instead of ethanolamine, collectively called plasmalogens, are found in animals and some anaerobic bacteria but not in plants, fungi or most aerobic bacteria except, notably, myxobacteria [23]. Human brain, heart and leukocytes are rich in plasmalogens, which occur in all subcellular membranes, and their deficiency or abnormal levels correlate with many disorders including cancer and Alzheimer’s disease [74,75,76]. As a result of their vinyl ether bond, plasmalogens can affect membrane fluidity and function, and have a proposed antioxidant role given their sensitivity to cleavage by ^1^O_2_ and other ROS [77]. However, plasmalogens had never been implicated in signaling photooxidative stress, a role that has now been clearly demonstrated in the *M. xanthus* light-induced carotenogenic response. Thus, deletion of *carF* annuls plasmalogen biosynthesis [23] as well as light-induced carotenogenesis [30,70], and the latter can be restored by supplying exogenous plasmalogens, even those from human cells that are distinct from the natural ones in *M.* *xanthus* (in that they have *sn*-1 and *sn*-2 moieties that differ from those in the *M. xanthus* VEPE). Furthermore, deleting genes (*elbD* and MXAN_1676) implicated in synthesis of the precursor AEPE (Figure 3c) impaired light-induced carotenogenesis, but was rescued by exogenous plasmalogen or by AEPE, which CarF converted to VEPE [23]. In sum, CarF is crucial in the response to light because it is indispensable for the biosynthesis of plasmalogens.

The role of plasmalogens in a blue light-PPIX-^1^O_2_ signaled response is both very recent and unprecedented, and identifying the underlying molecular mechanism of action is still being pursued. Breakage by ^1^O_2_ of the vinyl ether bond in the plasmalogen yields lyso-PE (2-monoacylglycerophosphoethanolamine) and a fatty aldehyde (Figure 3d; [23,77,78,79]). This may perturb local membrane structure, environment and properties and affect the function(s) of downstream effector(s) in the pathway. The cleavage products might also function as signaling lipids or second messengers to modulate (or inactivate) effector activity through establishing noncovalent interactions, or covalent adducts between the reactive fatty aldehyde product and target nucleophiles (lysines, cysteines or histidines in proteins). Plasmalogens may themselves bind to specific membrane proteins or complexes to directly modulate their functions through interactions with ^1^O_2_. These mechanisms, frequently invoked to link plasmalogens and cellular signaling [77,80], may also operate in *M. xanthus*.

### 7.3. Light-Induced Expression of the carQRS Operon and Gene crtIb

Early genetic analysis established that the *carR* locus encodes a negative regulator acting downstream of CarF [32,70], and that *carQ* and *carS*, closely linked to *carR*, encode positive regulators [33,34,35,36,37]. Subsequent DNA sequencing and transcription start site mapping revealed three translationally coupled genes, *carQ*, *carR* and *carS*, forming the *carQRS* operon and expressed from the light-inducible P_QRS_ promoter, which has –35 and –10 promoter elements divergent from typical *M. xanthus* RNAP-σ^A^ promoters [36]. Mutations at *carQ* are epistatic over those at *carR* and block activation of *carQRS* as well as of *crtIb*, the structural gene for carotenogenesis unlinked to the *carB* cluster [35,36,37,39]. Furthermore, *crtIb* expression is driven by a light-inducible promoter P_I_, with –35 and –10 promoter elements similar to P_QRS_ [39,81]. These findings therefore implicated CarQ in activating *carQRS* and *crtIb* expression from similar light-dependent promoters, and CarR in their downregulation.

**Figure 4:**
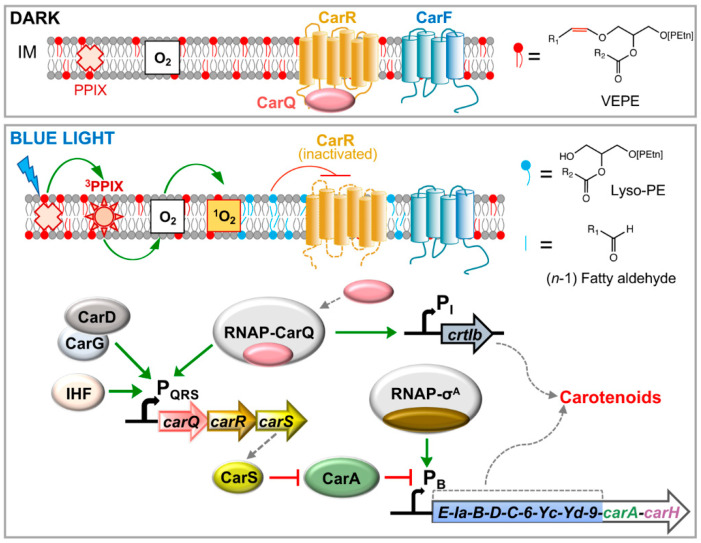
Model for the blue light-PPIX-^1^O_2_ signaling and transduction pathway, and regulation by the CarA-CarS repressor-antirepressor pair in *M. xanthus*. In the dark, the anti-σ factor CarR (with a six transmembrane-helix topology; IM: inner membrane) sequesters its cognate ECF-σ factor CarQ. Blue light excites PPIX to the high-energy ^3^PPIX state, from which energy transfer to molecular O_2_ generates the highly reactive ^1^O_2_. CarF produces plasmalogens (VEPE), which are required to transmit the ^1^O_2_ signal and cause the inactivation of CarR by a mechanism that remains to be elucidated. Plasmalogen cleavage by ^1^O_2_ might perturb the local membrane environment of CarR, or its cleavage products may interact with CarR, to alter its activity. This liberates CarQ, which associates with RNAP to activate promoters P_QRS_ (which also requires the CarD-CarG global regulatory complex and IHF) and P_I_ to drive expression of the regulatory *carQRS* operon and of the carotenogenic *crtIb* gene, respectively. CarS, expressed from P_QRS_ in the light, counteracts repression of P_B_ by CarA (see below) to drive expression of the *carB* operon, containing all but one of the carotenogenic genes, leading to the synthesis of carotenoids.

While CarS turned out to be the trans acting antirepressor of CarA [57,82], CarQ was identified as the founding member of a new, large and diverse group of alternative σ factors known as the extracytoplasmic function or ECF-σ factors, which were first discovered over 25 years ago [83,84]. Usually, ECF-σ act in a gamut of cellular responses to a variety of extracytoplasmic stimuli (hence the name) and are negatively regulated by association with cognate anti-σ factors, which are often membrane-bound and coexpressed with their ECF-σ partner [85]. CarR was shown to be such a membrane-bound anti-σ, as it specifically and stoichiometrically sequestered CarQ and rendered it inactive in the dark [37] through direct, physical interactions ([71,86]; Figure 4). With six transmembrane helices [36,86,87], CarR belongs to a small group of anti-σ factors with similar membrane topology, largely restricted to proteobacteria, and classified as DUF1109 in the conserved protein domain family database [85]. Some of these other anti-σ act in stress responses to ROS or to heavy metals [88,89,90] and, interestingly, transcription of both *carQRS* and *crtIb* is activated in the dark by copper [91]. The molecular basis for this copper-mediated action, which bypasses both CarF and light, is still unknown and remains to be elucidated.

CarQ must be first liberated from its cognate anti-σ CarR, which sequesters it in the dark [37], to associate with RNAP and initiate transcription of its target genes ([86]; Figure 4). Light triggers this liberation of CarQ from CarR, but the exact molecular mechanism remains elusive. CarR was reportedly unstable when exposed to light, especially when cells enter the stationary phase of growth [86], which also correlates with PPIX accumulation. Increased PPIX levels, however, do not activate P_QRS_ and *carQRS* expression in the absence of CarF [30], and since the actual role of CarF is in plasmalogen synthesis, this lipid must somehow mediate inactivation of CarR by light [23]. Various mechanisms, as noted before, can be hypothesized for how plasmalogens mediate CarR activation. Plasmalogen cleavage by ^1^O_2_ might perturb the local membrane environment of CarR, or a cleavage product may interact with CarR to alter its activity. Other unknown player(s) or mechanism(s) cannot also be ruled out. These questions will have to be resolved in future work.

### 7.4. Regulation of CarQ Activity in Light-Induced Expression of carQRS and crtIb

Negative regulation by CarR is the key determinant of CarQ activity since this controls its availability for association with RNAP. Nonetheless, additional factors required for CarQ activity have also been discovered. An early screen of Tn*5*-*lac* Car^−^ mutants identified two constitutively expressed genes, *carD* and *ihfA*, acting directly in light-induced activation of *carQRS* and, through expression of CarQ and CarS, indirectly in those of *crtIb* and the *carB* operon, respectively [92,93]. The *ihfA* gene encodes the α subunit of the integration host factor (IHF) heterodimer, a nucleoid-associated, histone-like architectural factor that functions as a global regulator [94,95]. Gene *carD* encodes a 316-residue DNA-binding transcriptional factor and is translationally coupled to a downstream gene, *carG*, whose product forms with CarD a tight heteromeric complex that functions as one regulatory unit ([96,97,98]; Figure 4). CarG is therefore essential for CarD function and the two always coexist. Interestingly, the pair occurs exclusively in *M. xanthus* and related myxobacteria. Thus, at least three other proteins besides CarR, namely IHF, CarD and CarG, regulate CarQ activity at P_QRS_.

**Figure 5:**
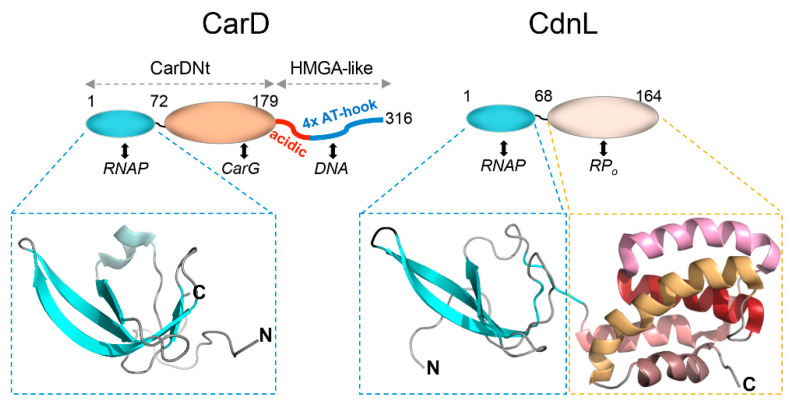
*M. xanthus* CarD and CdnL. Schematics summarizing structural and functional domains of *M. xanthus* CarD (left) and CdnL (right). Numbers correspond to the residues delimiting the indicated domains, which interact with the partners listed below. Bottom: Structures determined for the RNAP interacting module of CarD (PDB accession code 2LT1) and full-length CdnL (PDB accession code 2LWJ).

Both CarD and CarG are unusual transcription factors. CarG is a monomer with no DNA-binding capacity, which coordinates two zinc atoms via a His-Cys rich segment (HQx_2_Hx_2_Ex_2_HCx_4_CxMx_16_Cx_2_C; x is any amino acid) [96]. The motif is similar to one found in zinc-metalloproteases called metzincins [99] but an E essential for protease activity is replaced by Q in the motif in CarG, which has no protease activity [96]. In short, CarG can be considered to be one more among the few bacterial transcriptional factors that do not bind DNA [100,101], but which appears exclusively in myxobacteria. CarD is also a rather singular protein. One striking feature is its ~136-residue C-terminal segment comprising a highly acidic ~50-residue region flanked by a C-terminal segment containing four repeats of the RGRP “AT-hook” DNA-binding motif (Figure 5; [102]). Interestingly, these motifs are rare in bacteria but occur in eukaryotic proteins such as high-mobility group type A (HMGA), a relatively abundant, nonhistone architectural factor that remodels chromatin in various DNA transactions [97,102,103,104]. Similar to HMGA, the CarD C-terminal domain is intrinsically disordered and binds to the minor groove of appropriately spaced AT-rich DNA tracts; and two such tracts at P_QRS_ (at –63 and –77 relative to the transcription start site) to which CarD binds are implicated in CarQ activity [96,98,103,105]. In line with this, the minimum P_QRS_ segment required for CarQ activity is a ~145 bp upstream stretch starting from the transcription start site [106]. By comparison, CarQ activity at its other target promoter, P_I_, which CarD and IHF affect indirectly, requires a shorter stretch extending to position –54 upstream of the transcription start site [81]. Interestingly, CarD can function in *M. xanthus* even when its natural HMGA-like domain is replaced by human HMGA, histone H1 or the intrinsically disordered H1 C-terminal region, indicating that a basic, structurally disordered C-terminal domain is sufficient for CarD function [98]. Surprisingly, even without its HMGA-like domain, CarD functions in vivo, albeit with diminished activity [107]. By contrast, the remaining N-terminal region of CarD is indispensable for function [108].

Unlike its intrinsically disordered HMGA-like C-terminal domain, the CarD N-terminal domain, CarDNt (Figure 5), is a structurally defined module with sequence similarity to the RNAP-binding domain of bacterial transcription repair coupling factors or TRCFs [103], which repair lesions in the transcribed strand by interacting with RNAP [109]. Indeed, CarDNt has an N-terminal subdomain with a five-stranded β-sheet Tudor-like tertiary structure (Figure 5) similar to its counterpart in TRCF [107] and interacts specifically with the RNAP β subunit [105,110]. Moreover, the C-terminal part of CarDNt, not involved in the interaction with RNAP, binds to CarG [96,98,107]. CarDNt is thus a protein–protein interaction hub directing interactions with both RNAP and CarG, while the CarD HMGA-like C-terminal domain mediates DNA binding.

Remarkably, although CarD homologs are restricted to myxobacteria closely related to *M. xanthus*, CarDNt is a defining member of a large family of bacterial RNAP-interacting proteins (PF02559 or CarD_CdnL_TRCF protein family; http://pfam.sanger.ac.uk, accessed on 29 April 2021) that includes not only CarD and TRCF homologs but also a large group of standalone proteins similar to CarD without its HMGA-like domain. These proteins, denoted CdnL (for CarD N-terminal Like), are widely distributed in bacteria and occur in *M. xanthus* and other δ-proteobacteria, α-proteobacteria, Actinomycetes, Firmicutes, Deinococcus-Thermus and Spirochaetes, but not in β-, γ- or ε-proteobacteria, Chlamydiae or Cyanobacteria [108,110,111]. Whereas knocking out *carD* does not affect normal growth or viability, CdnL is indispensable for normal growth and survival of *M. xanthus* [110,112,113]. CdnL has also been reported to be an essential gene in *Borrelia burgdorferi* (spirochaetes), *Mycobacterium tuberculosis* (mycobacteria) and *Rhodobacter sphaeroides* (α-proteobacteria), and to impair normal growth in *Caulobacter crescentus* (α-proteobacteria) [111,114,115,116]. This is because transcription of the essential RNAP-σ^A^-dependent rRNA genes in these bacteria requires CdnL in the critical step of open promoter complex (RP_o_) formation, and CdnL directly or indirectly impacts expression of important biosynthetic genes [111,113,116,117,118]. In contrast to CarD, which interacts with both RNAP and CarG via CarDNt [105,107], CdnL interacts only with RNAP [110] and does not bind DNA, since it lacks the HMGA-like domain [113]. Nevertheless, the CdnL N-terminal region conserves both the structure and contacts with the RNAP β subunit of its equivalent in CarDNt (Figure 5; [107,113]). When associated to RNAP in RP_o_, the compact C-terminal region of CdnL can interact with promoter DNA from positions –14 to –10 to stabilize the transcription bubble [101,117]. Some of the functionally important residues in this CdnL domain, which comprises five well-packed α-helices (Figure 5; [113]), are conserved and important for CarD function as well, and mutating these affects CarD function at target promoters even though binding to CarG remains unaffected [107].

Importantly, the CarD-CarG complex affects processes other than light-induced carotenogenesis in *M. xanthus*. It was implicated in regulating the expression of some early genes in the starvation-induced development to multicellular fruiting bodies [92,96] and of various vegetatively expressed genes of mostly unknown functions [119], none CarQ-dependent. A later study showed that the CarD-CarG complex affects the activities of at least twelve ECF-σ/anti-σ pairs besides CarQ-CarR in *M. xanthus*, suggesting that the complex may control many of the ∼45 putative ECF-σ factors in this bacterium [87]. Except for the light-induced CarQ-CarR pair, the signals that activate each of the other ECF-σ/anti-σ pairs that depend on CarD-CarG are unknown. One pair was, however, recently shown to direct the expression of one of the three CRISPR-Cas systems (type III-B) in *M. xanthus*, which suggested that this bacterial defense system is triggered by a phage [120]. Thus, CarD-CarG is a global regulator, like CdnL, but targets different genes. The parallels with CdnL (despite differences) and the finding that the CarD-CarG complex targets various ECF-σ promoters suggests that CarD-CarG may have a role at these promoters analogous to that of CdnL at RNAP-σ^A^-dependent promoters [107]. This remains to be further explored in future studies.

### 7.5. Derepression of P_B_ by Light-Induced Expression of the CarS Antirepressor

**Figure 6:**
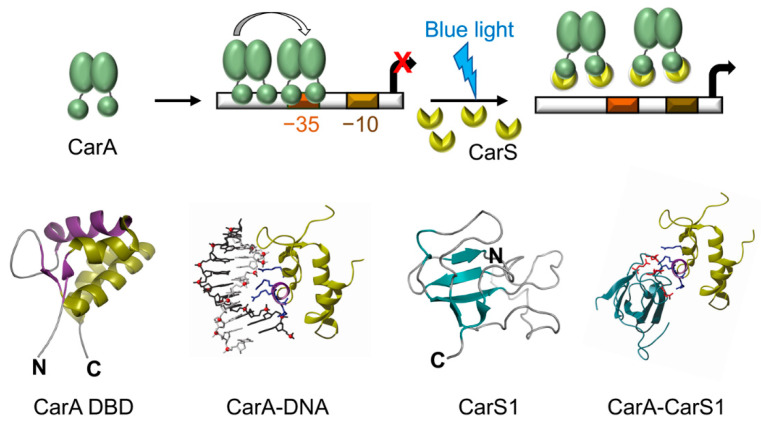
Molecular mechanism of CarA-CarS repressor-antirepressor mode of regulation at P_B_. In the dark, CarA dimers bind cooperatively to its operator at P_B_, which blocks access to RNAP-σ^A^ and represses transcription. CarS, expressed from the *carQRS* operon in the light, acts as a DNA mimic to sequester the CarA DBD and prevent its binding to operator, thereby enabling transcription initiation by RNAP-σ^A^ at P_B_. Bottom: structures of the CarA DBD and CarS1 (PDB accession codes 2JML and 2KSS, respectively) and structural models for CarA-DNA and CarA-CarS1 complexes.

The photoregulatory switch controlling expression of the *carB* cluster from the P_B_ promoter relies on repression by both CarH and CarA, and their inactivation by light. Whereas CarH is a photoreceptor that directly senses light, CarA repression is relieved by physical interaction with the CarS antirepressor, whose expression is induced by light (Figure 6; [56,57,58,59,82,121]). A series of biochemical, structural and mutational studies demonstrated that the CarA N-terminal domain is an autonomous folding unit with the winged-helix topology of MerR family DBDs (Figure 6), and that it contains the determinants for specific binding to operator DNA as well as to CarS [57,58,59]. Further structural-mutational analysis revealed that the highly acidic, 111-residue CarS adopts a five-stranded, antiparallel β-sheet fold resembling SH3 domains (protein–protein interaction modules prevalent in eukaryotes but rare in prokaryotes) and contains a solvent-exposed hydrophobic pocket lined by acidic residues that mimics operator DNA to bind tightly to the DNA recognition helix of CarA and sequester it (Figure 6; [121]). Interestingly, a gain-of-function *carS* mutant (*carS1*) lacking the 25 C-terminal residues results in constitutive, light-independent expression at P_B_ [36], presumably because the variant CarS1 is more acidic than CarS and thus binds more tightly to CarA [57]. Given that CarH recognizes the same operator as CarA and both proteins have similar DBDs and recognition helices, CarH also physically interacts with CarS, albeit with lower affinity than CarA [20,60,121]. Thus, repression of P_B_ by CarA is counteracted by CarS expressed only under light, while P_B_ repression by CarH is relieved mostly by the direct effect of light on the AdoCbl chromophore. CarS homologs occur only in myxobacteria related to *M. xanthus*, and likely play an analogous antirepressor role.

## 8. Conclusions

Delving into how *M. xanthus* “sees” and mounts a photooxidative stress response that triggers carotenogenesis uncovered two novel pathways in bacterial light sensing, signal transduction and gene regulation. One pathway relies on a form of vitamin B_12_ and its association with a single photoreceptor-cum-transcriptional factor, and the other is a B_12_-independent, more complex route that requires various singular factors. Many worthy firsts can be credited to elucidation of the two pathways, including the discovery of one of the first ECF-σ factors, CarQ [83,84]; the founding members of large protein families, notably the B_12_-based CarH photoreceptor family [19,20,21,22] and the CarD_CdnL family of RNAP-binding transcription factors [102,103,110]; the long-sought human desaturase involved in plasmalogen biosynthesis through its *M. xanthus* CarF homolog [23]. Insights specific to *M. xanthus* and closely related bacteria, but also ones more broadly conserved across bacteria, have emerged. This photooxidative stress response is linked, directly or indirectly, to that of copper and to heme and fatty acid biosynthesis, and shares global regulators with processes as diverse as fruiting body development and activation of CRISPR-Cas systems. Future work will undoubtedly reveal new, possibly surprising, interconnections to other cellular activities.

Beyond bacterial physiology, signaling and gene regulation, the findings from *M. xanthus* light-induced carotenogenesis have had other important ramifications. How this response and its unique factors are conserved across bacteria and other organisms provides valuable evolutionary insights. Some of the factors involved, which are more typical of eukaryotes, yield phylogenetic signals that may be supportive of the hypothesis that an ancient myxobacterium may have contributed in eukaryogenesis [122]. This hypothesis, known as the Syntrophy hypothesis for the origin of eukaryotes, posits that the eukaryotic cell evolved from symbiosis or syntrophy between a complex early myxobacterial-like deltaproteobacterium (host), an endosymbiotic Asgard-like archaeon (future nucleus) and an alphaproteobacterium (future mitochondrion) [122]. The role of a myxobacterium proposed in this hypothesis was based on the many myxobacterial-like genes in eukaryotes. These phylogenetic signals include, among various others, isoprenoid biosynthesis enzymes, HMGA proteins (CarD) [122], CarF and plasmalogens [23].

Satisfyingly, CarH has now been exploited as one of the few green-light responsive optogenetic tools for light-controlled: (a) gene expression in *M.* *xanthus* and transgene expression in mammalian and plant cells; (b) receptor interactions and signaling in human cells and zebra fish embryos; (c) generation of protein hydrogels that enable facile encapsulation and release of cells and proteins, and cell adhesions [123,124,125,126,127,128]. Notably, this last application was very recently adapted to address challenges in regenerative neurobiology to engineer metal-coordinated protein hydrogels for sustained delivery of neuroprotective cytokines aimed at neuronal survival and axon regeneration in vivo [126].

The discovery that CarF and its human and animal homologs are identical lipid desaturases essential in plasmalogen synthesis has not only revealed a remarkable conservation of this enzyme across a vast evolutionary distance, but also has important implications in human health and disease [23]. Plasmalogens have been linked to various human disorders including cancer and Alhzeimer´s disease but the unknown identity of plasmanylethanolamine desaturase had been an impediment in directly assessing the role of these lipids in diverse pathologies. This is now possible with the identity of the enzyme in hand, and has already proved useful in studies of mitochondrial metabolism [129] and ferroptosis [130,131].

## Data Availability

Not applicable.
